# Supplementary material for: SNP associations in the L-citrulline metabolic pathway and vascular aging in the Japanese population
Source: PLoS One. 2025 May 29;20(5):e0323778. doi: 10.1371/journal.pone.0323778 (PMC12121766; doi:10.1371/journal.pone.0323778)

Mood disturbance

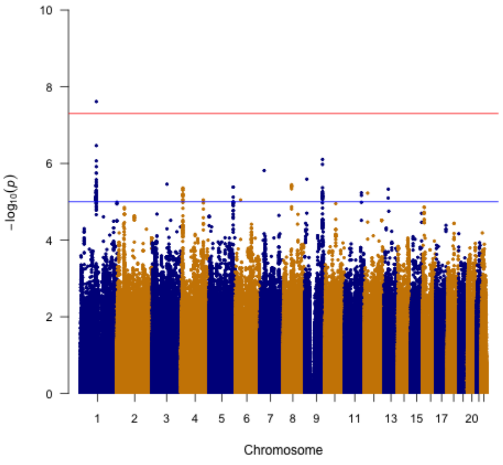

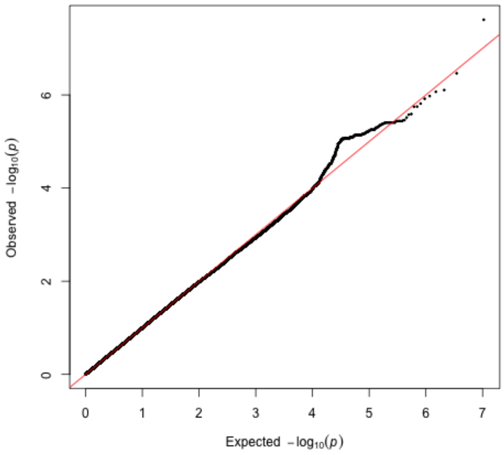

Anger

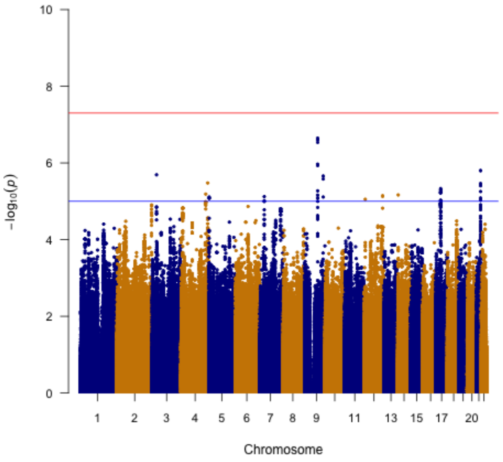

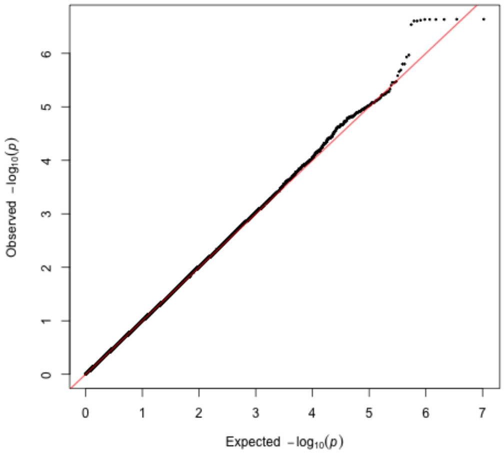

Confusion

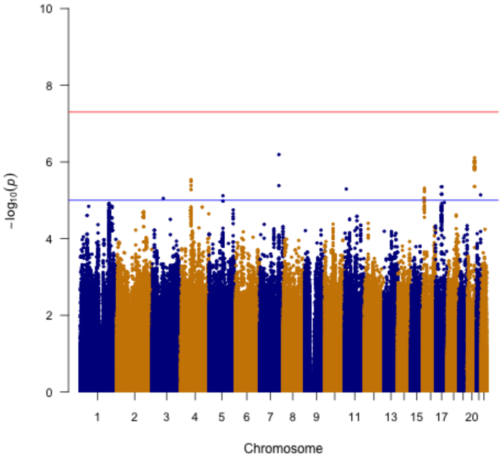

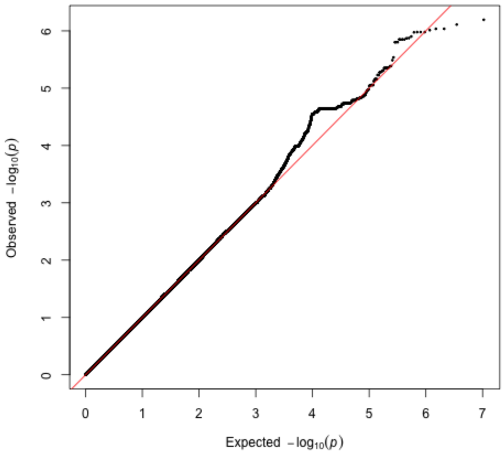

Depression

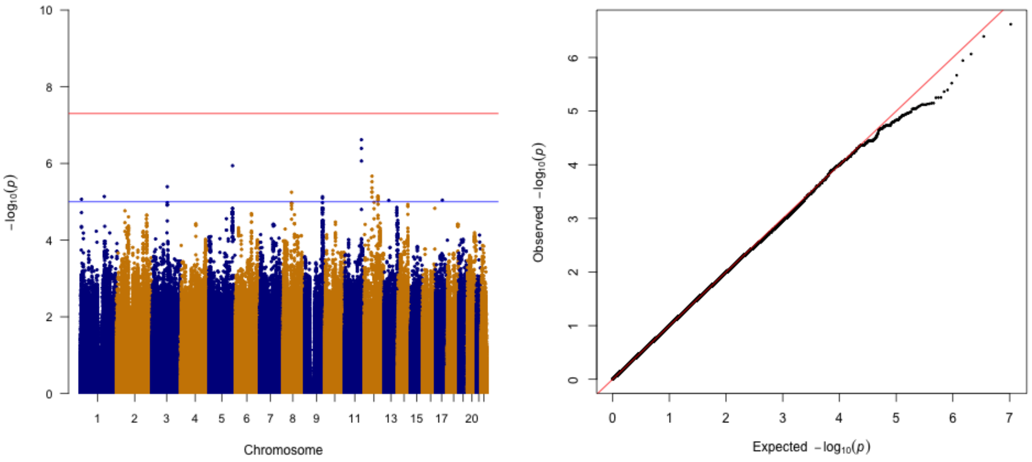

Fatigue

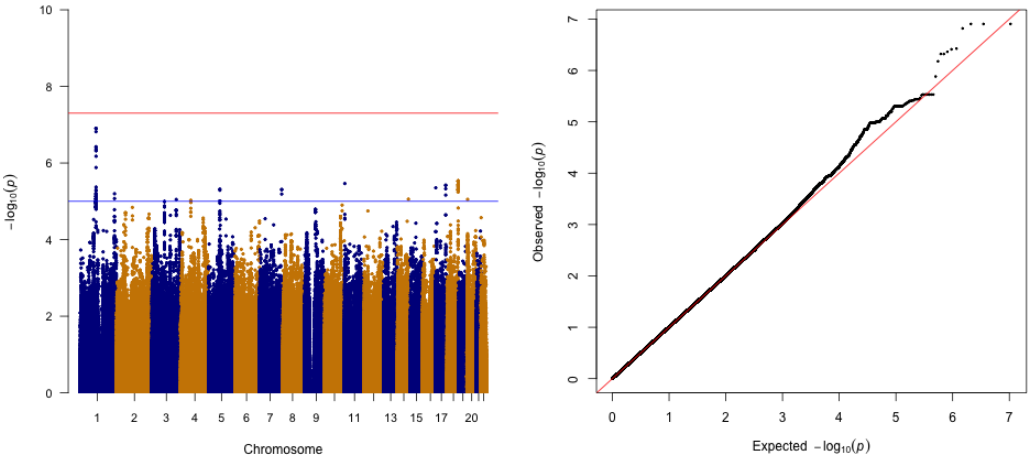

Tension

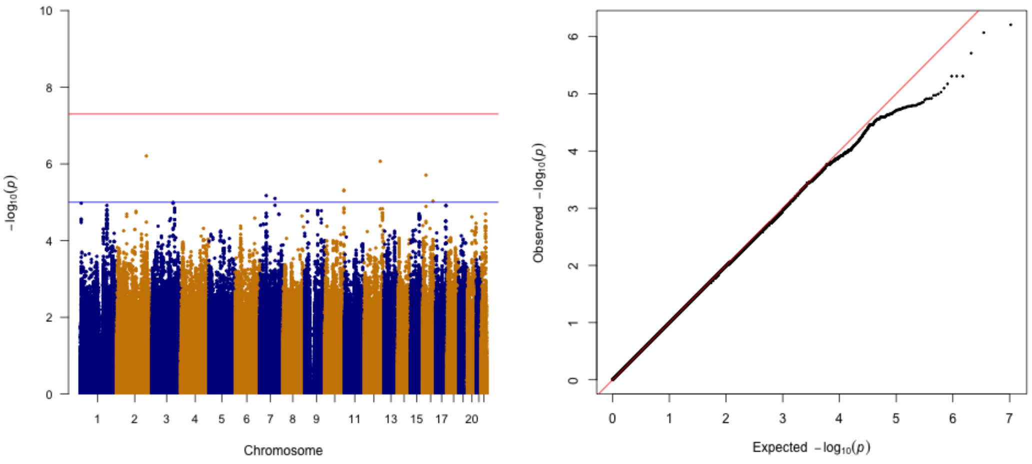

Lethargy

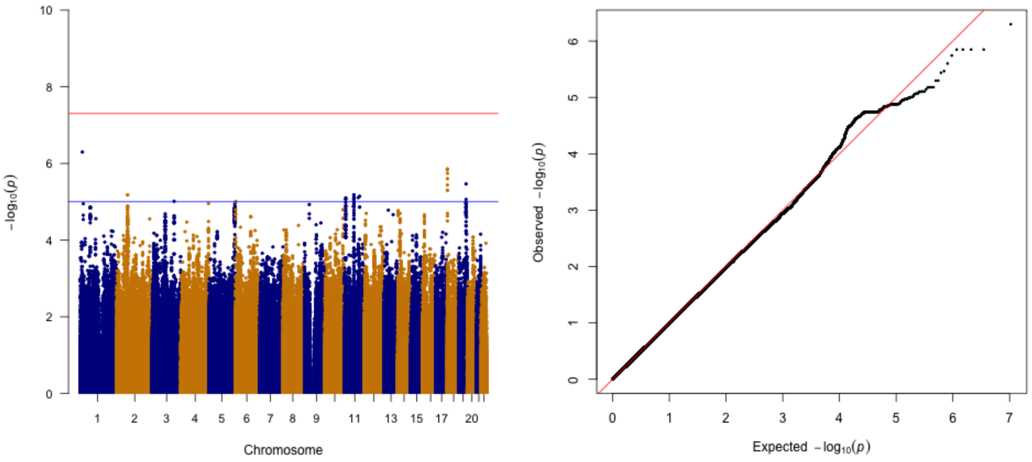

Unfriendliness

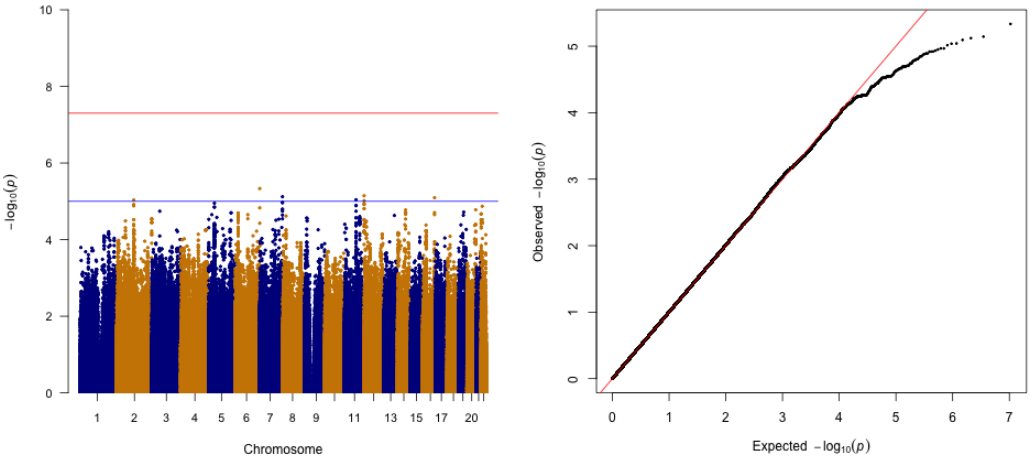

Poor physical functioning

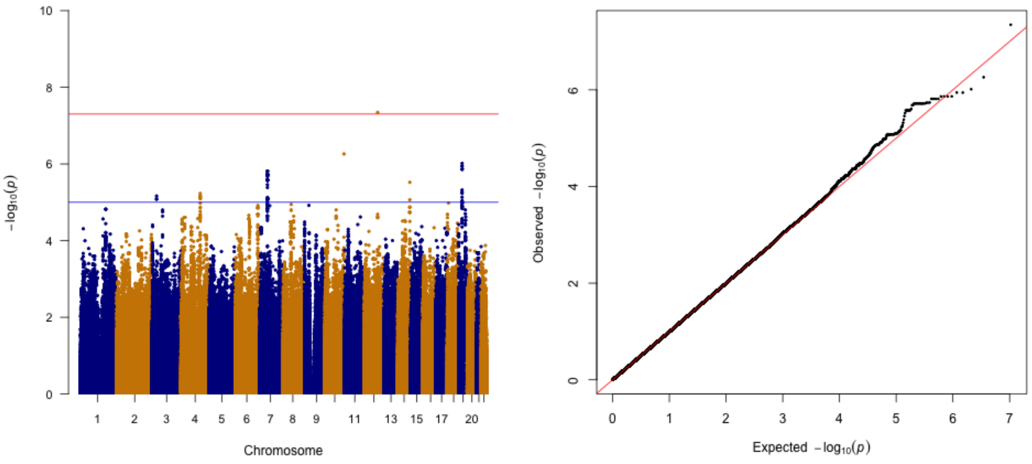

Poor role physical

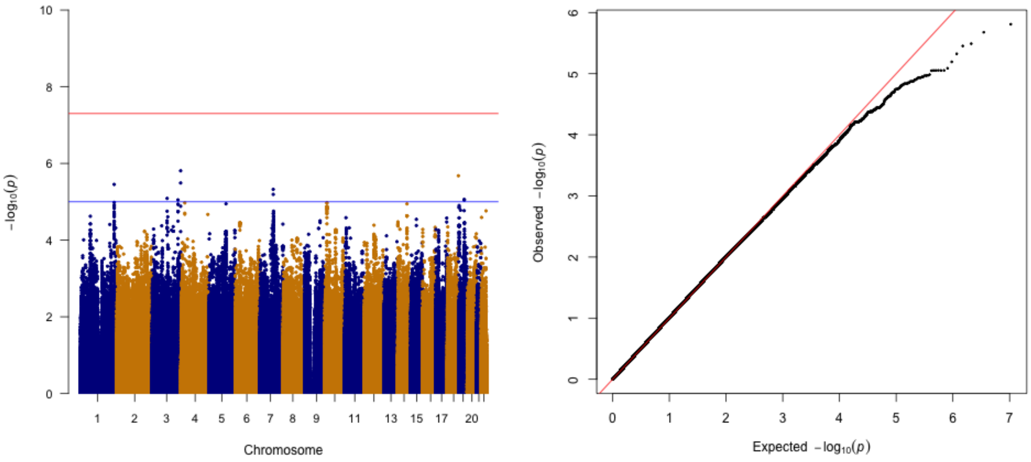

Body pain

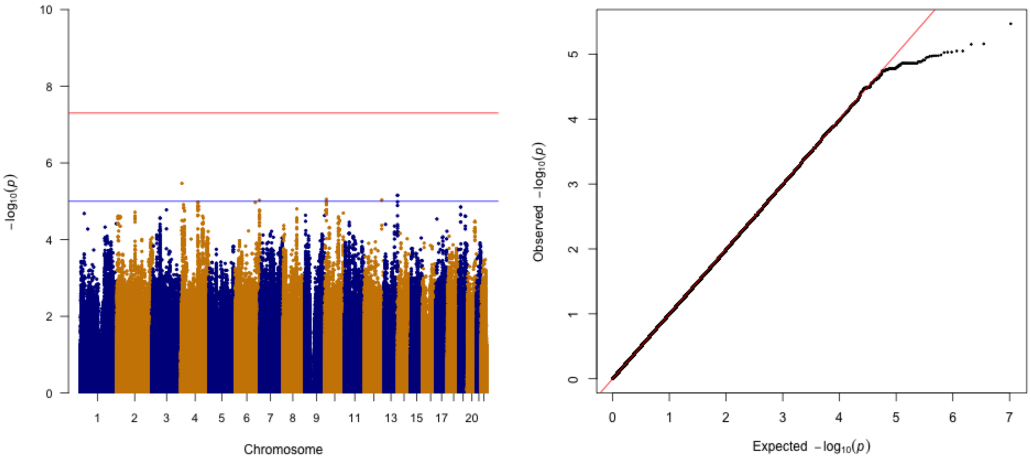

Poor general health

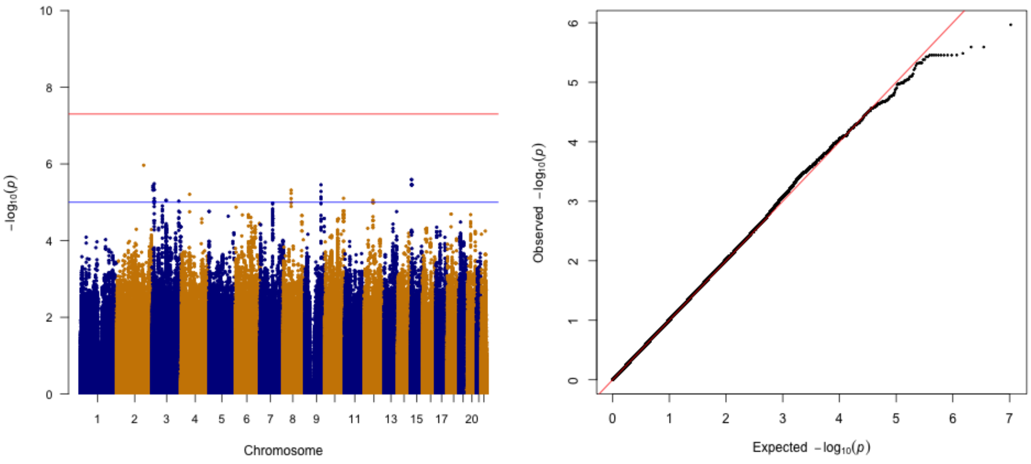

Poor vitality

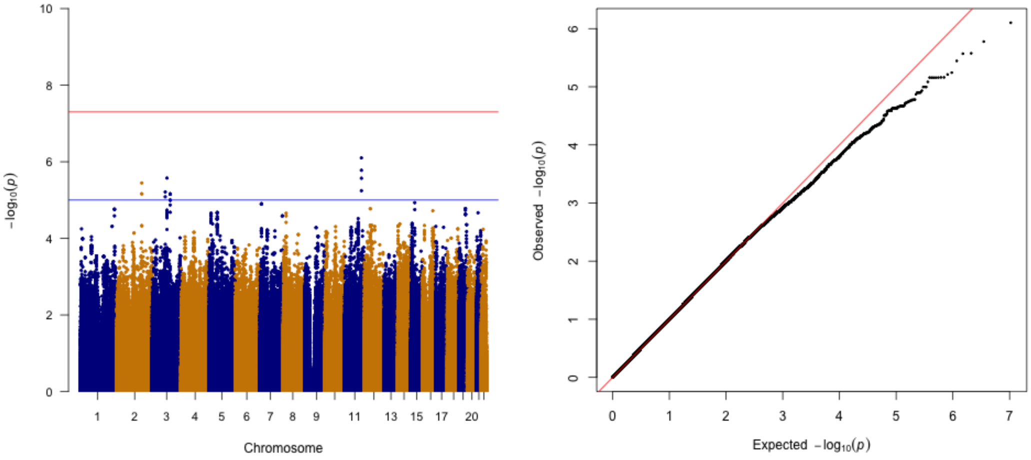

Poor social functioning

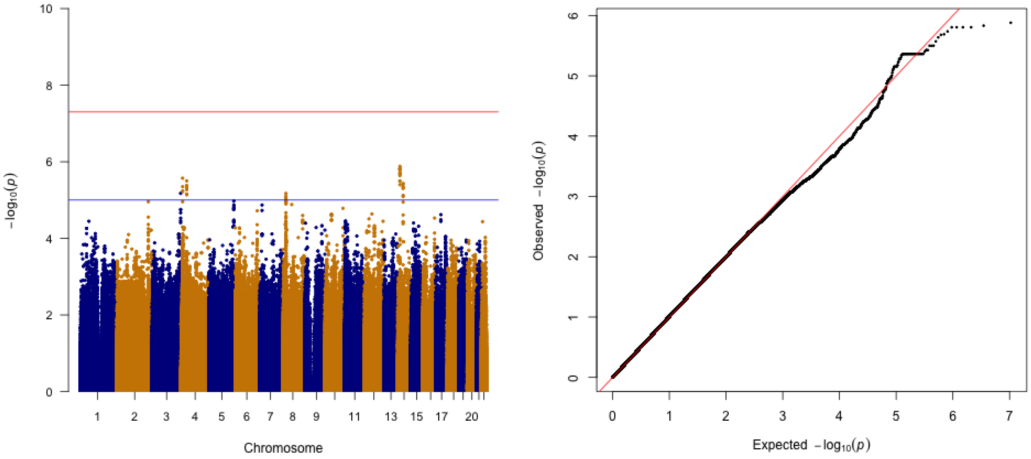

Role limitations due to emotional problems

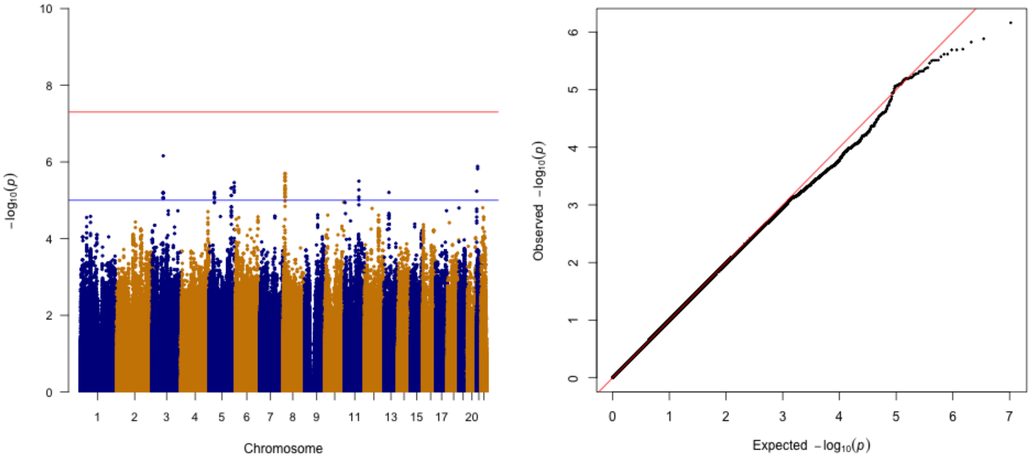

Poor mental health

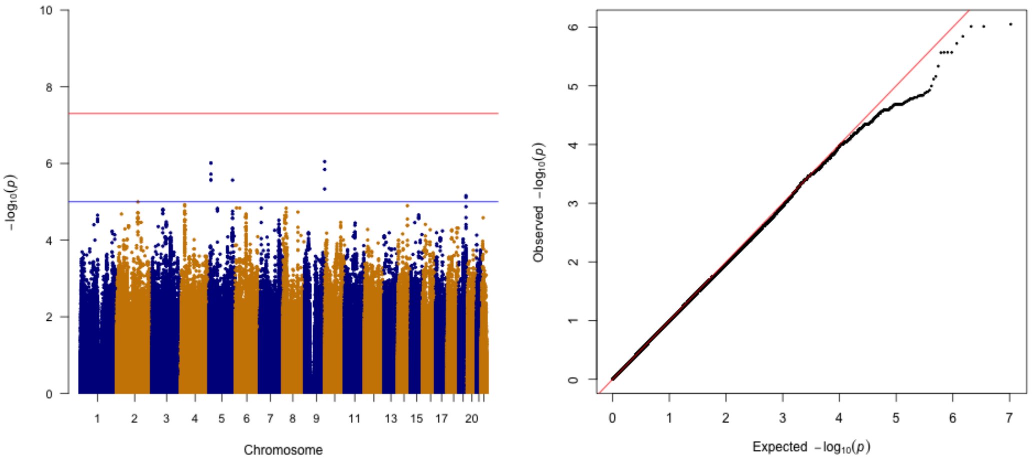

Poor physical functioning with negative mood states

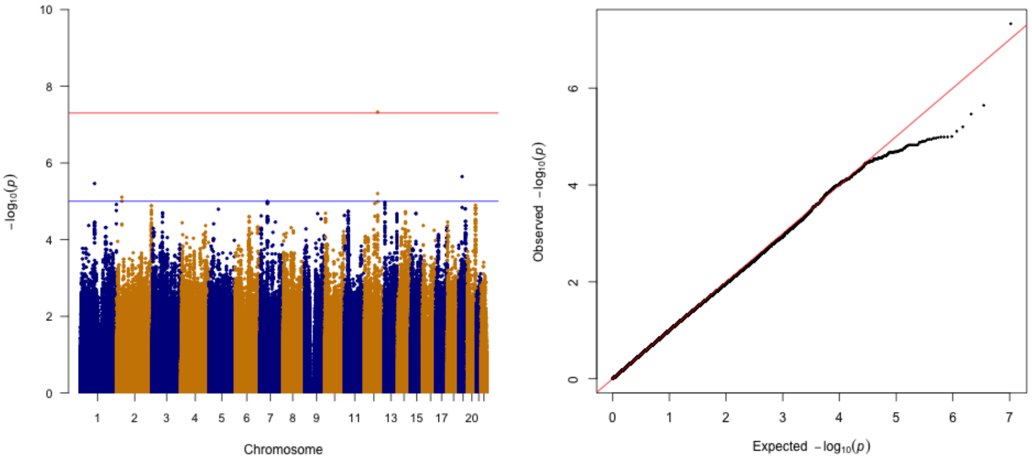

Poor role physical with negative mood states

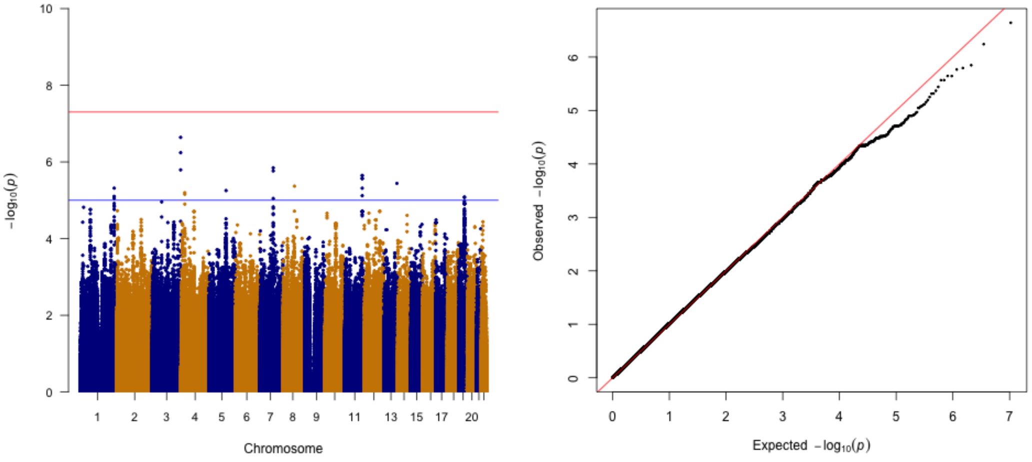

Body pain with negative mood states

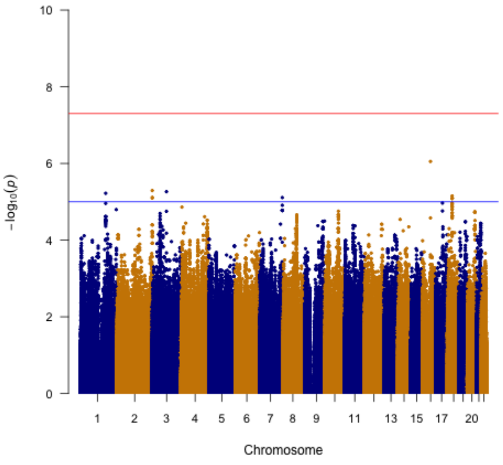

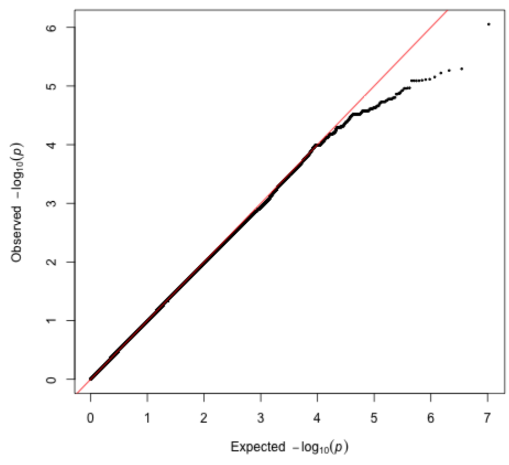

Poor general health with negative mood states

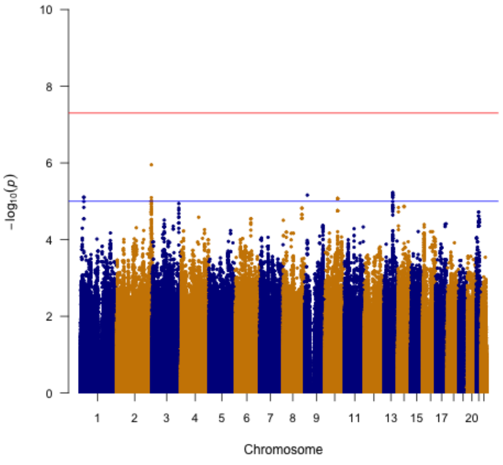

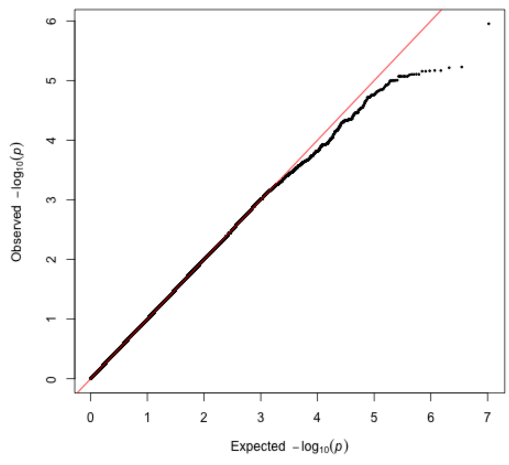

Poor vitality with negative mood states

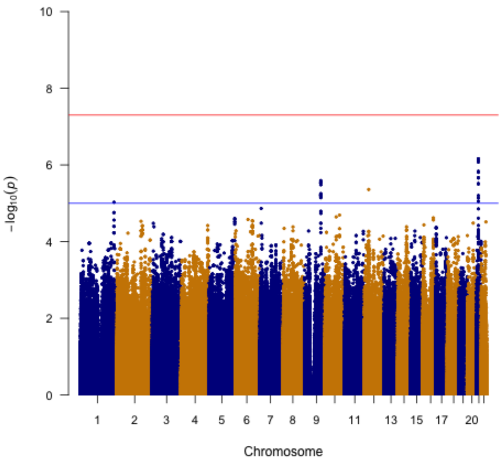

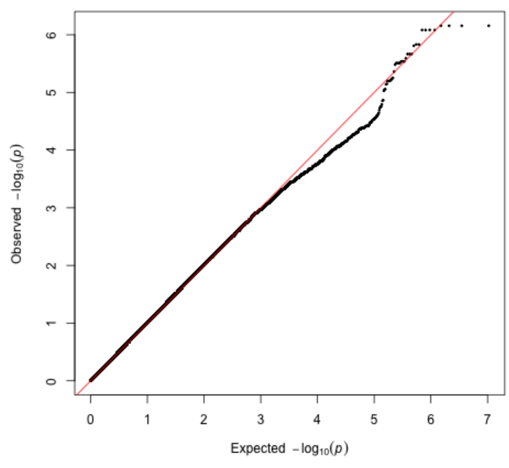

Poor social functioning with negative mood states

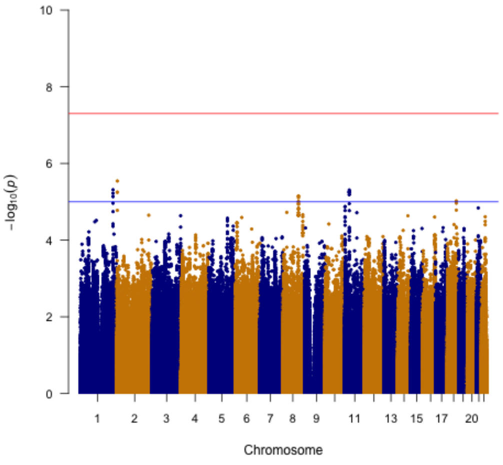

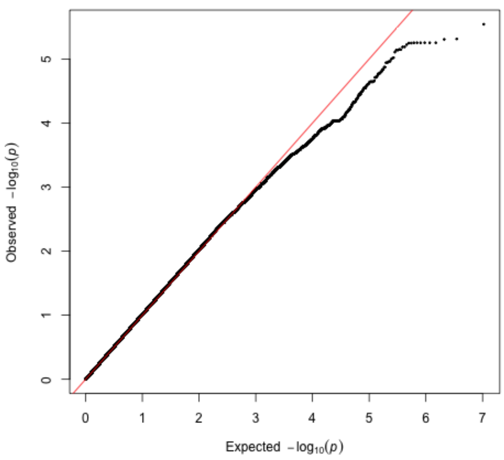

Role limitations due to emotional problems with negative mood states

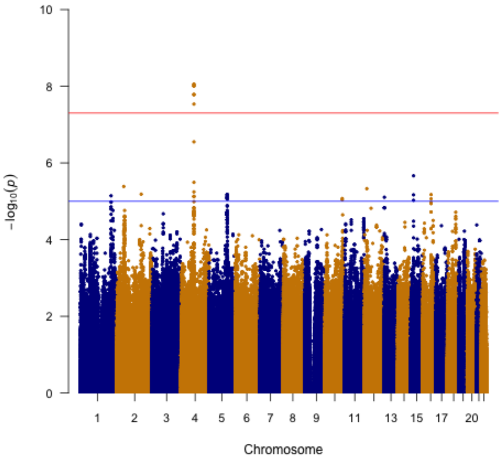

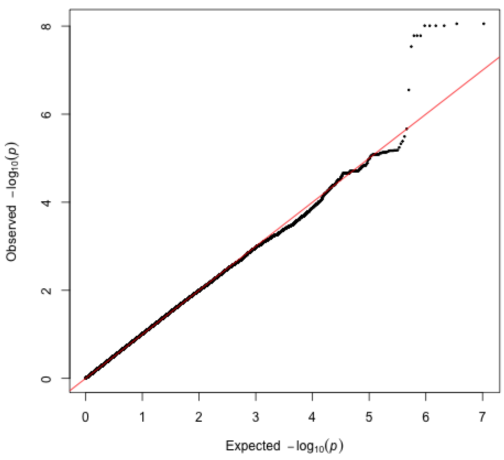

Poor mental health with negative mood states

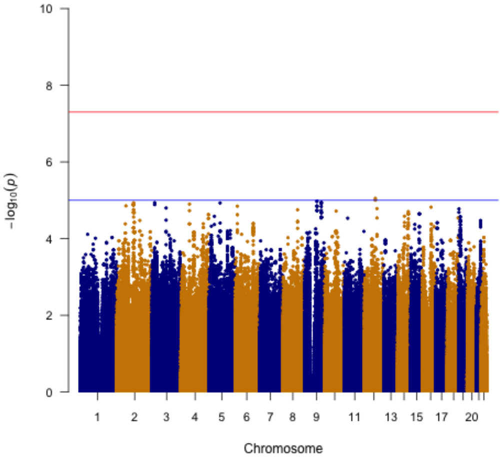

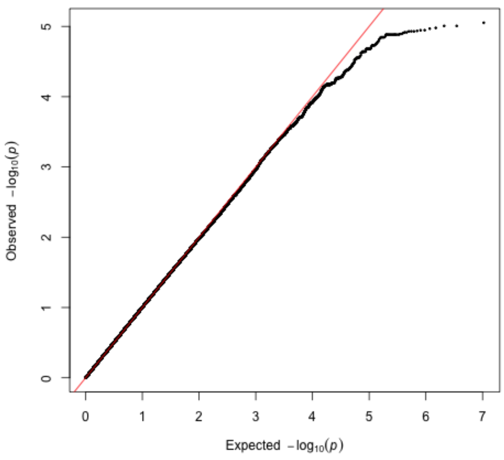

Cold body

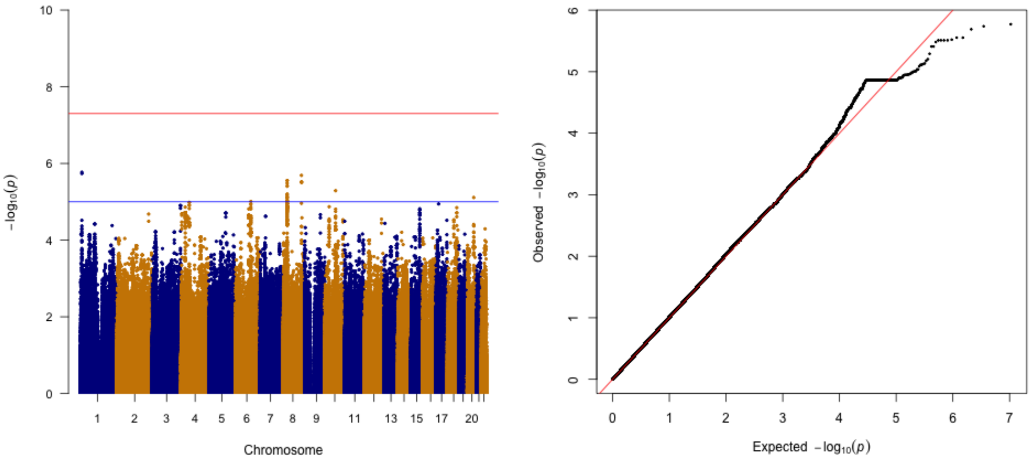

Cold hands and feet

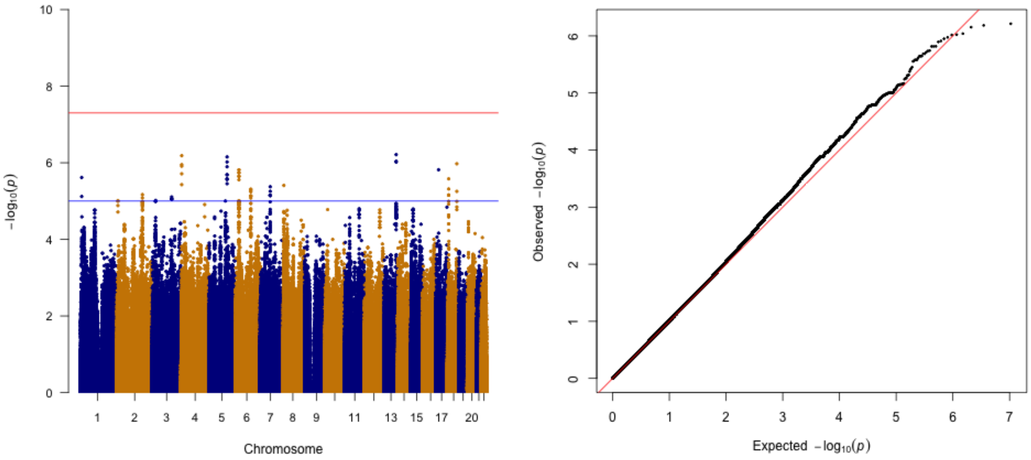

Swelling hands and feet

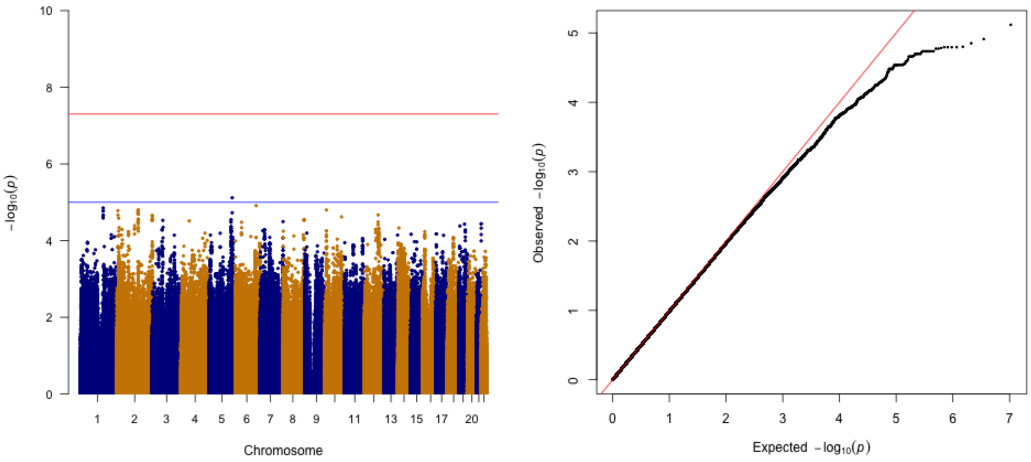

Warm body

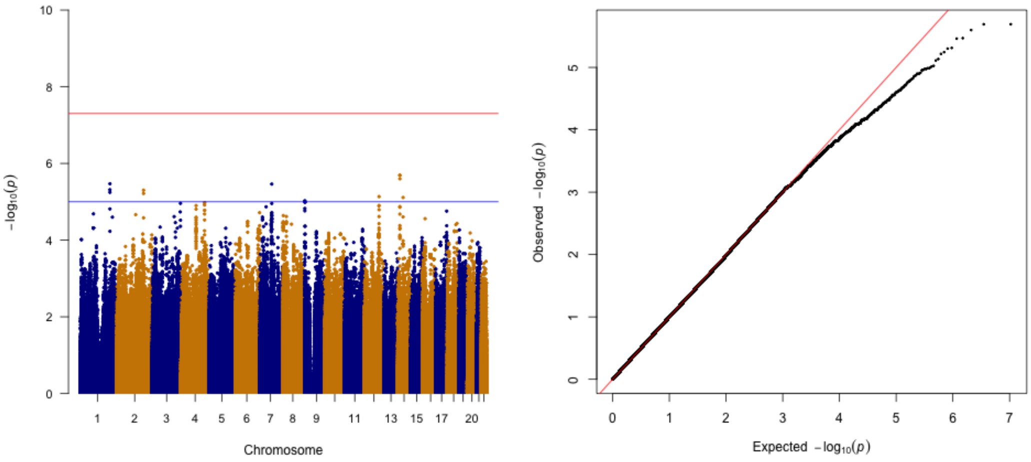

Pale face

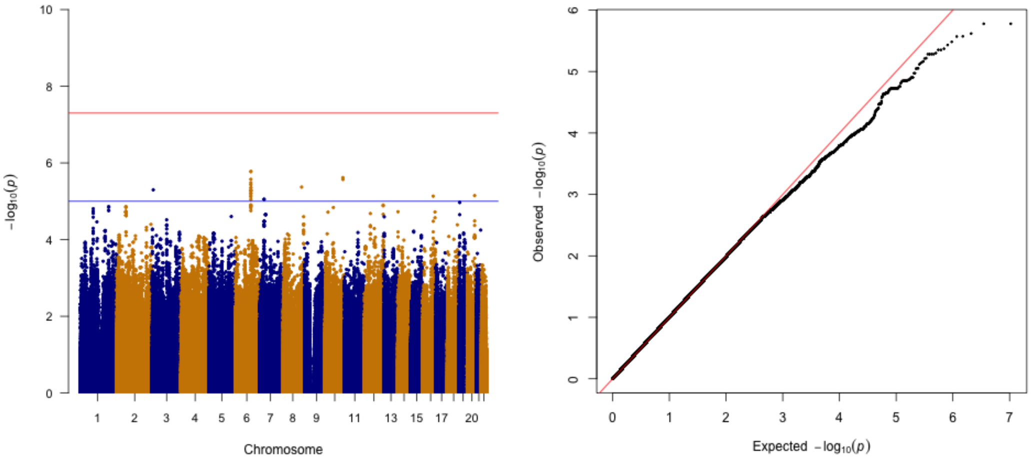

Decreased skin elasticity

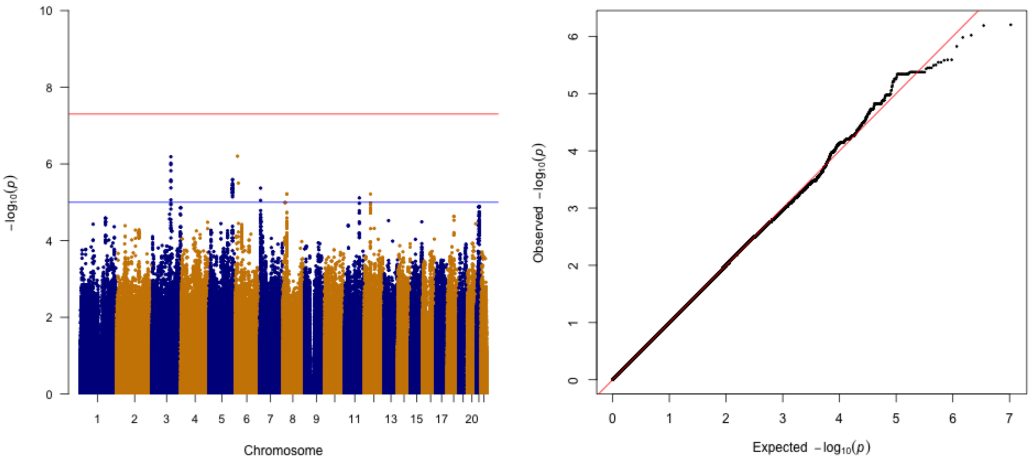

Dizziness

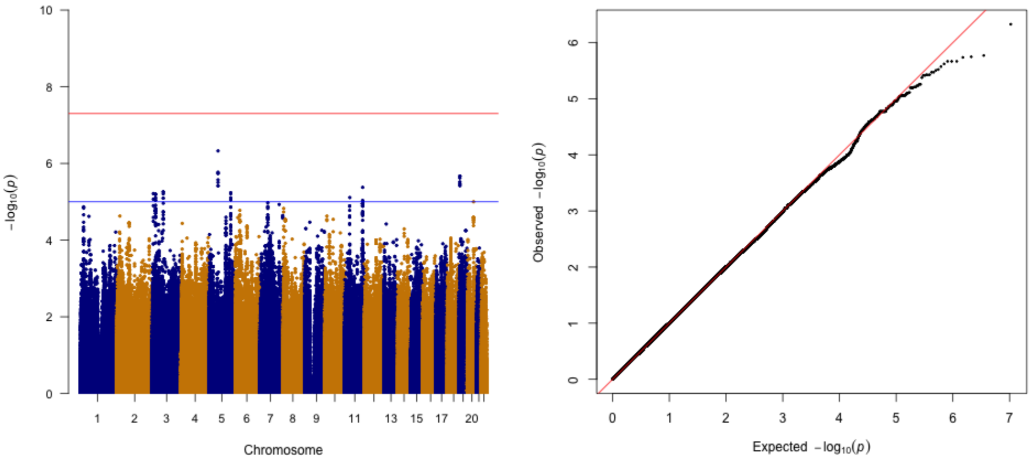

Numbness of limbs

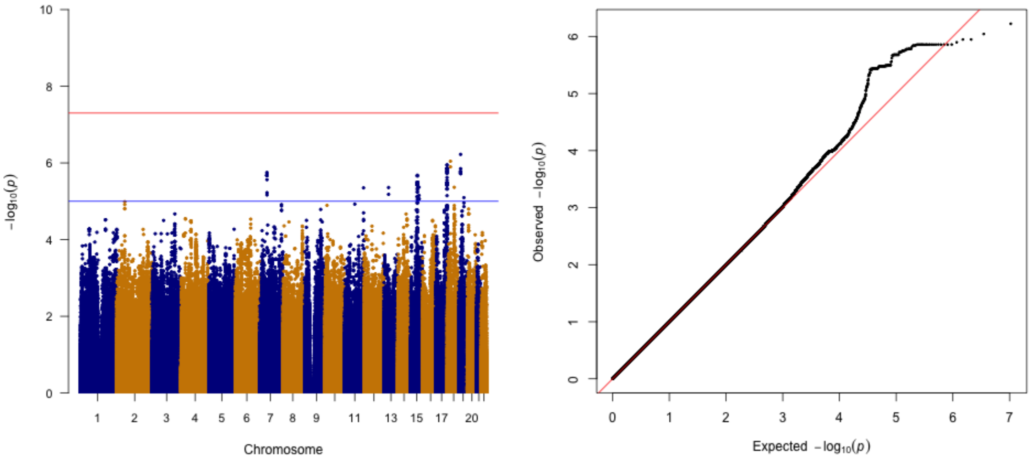

Tired easily

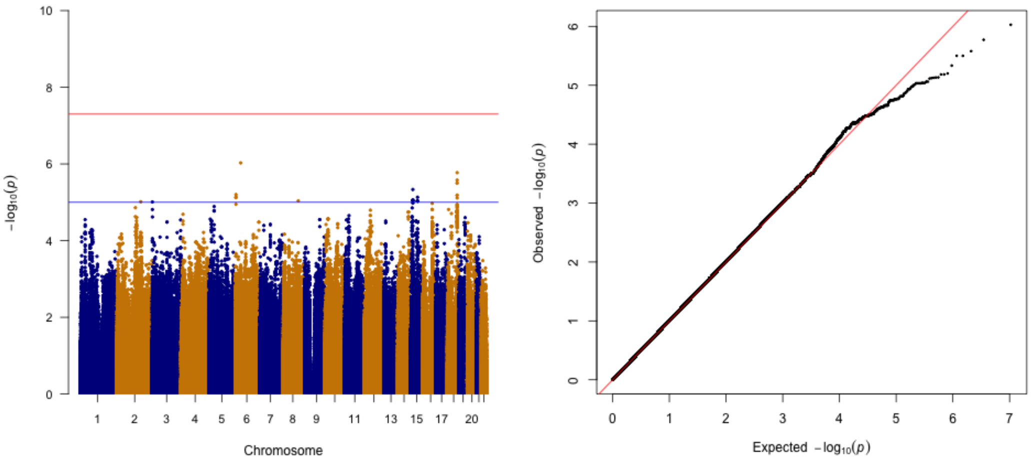

Eye fatigue

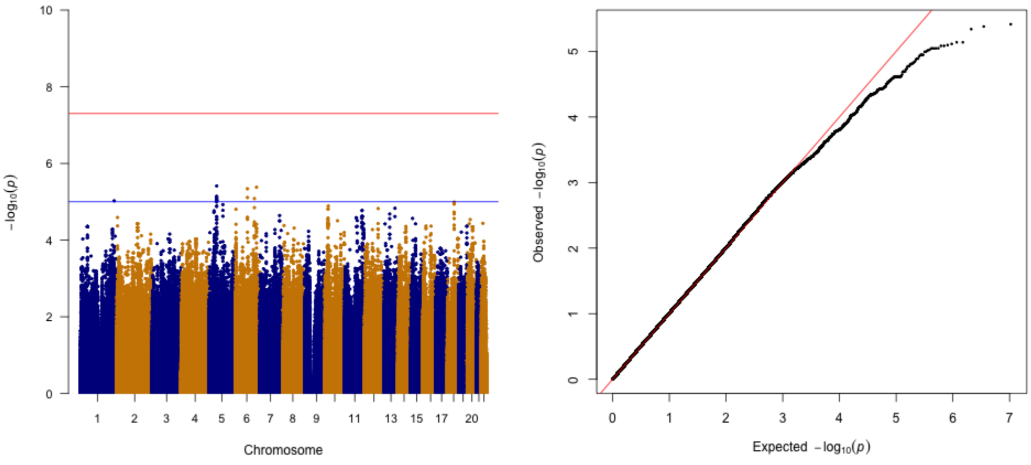

Bad awakening

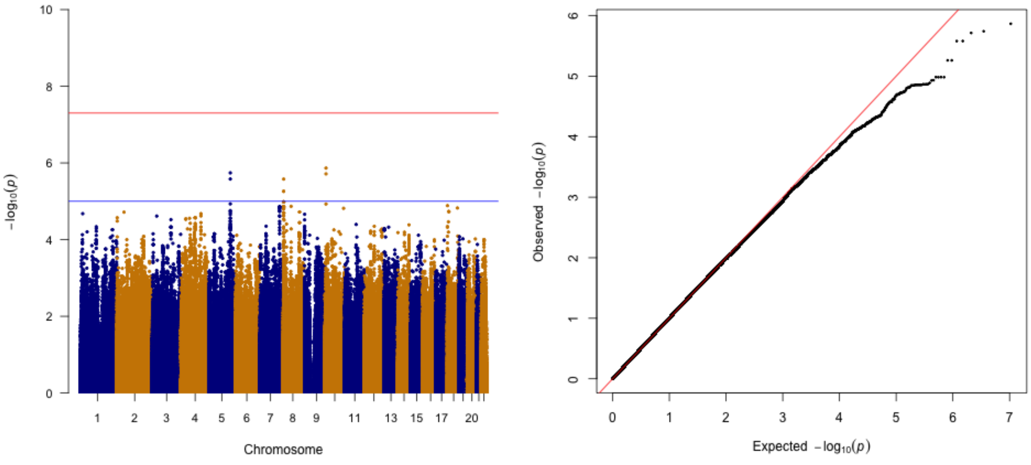

Difficulty in getting sleep

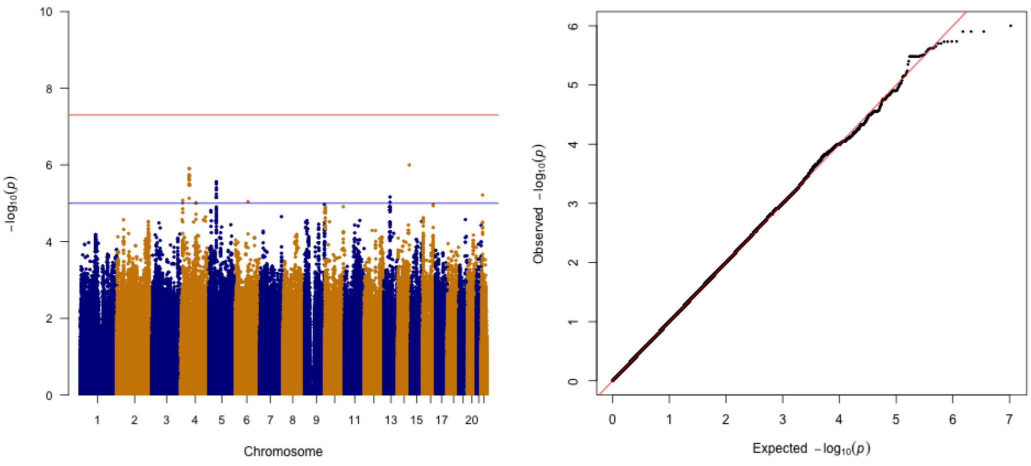

Light sleep

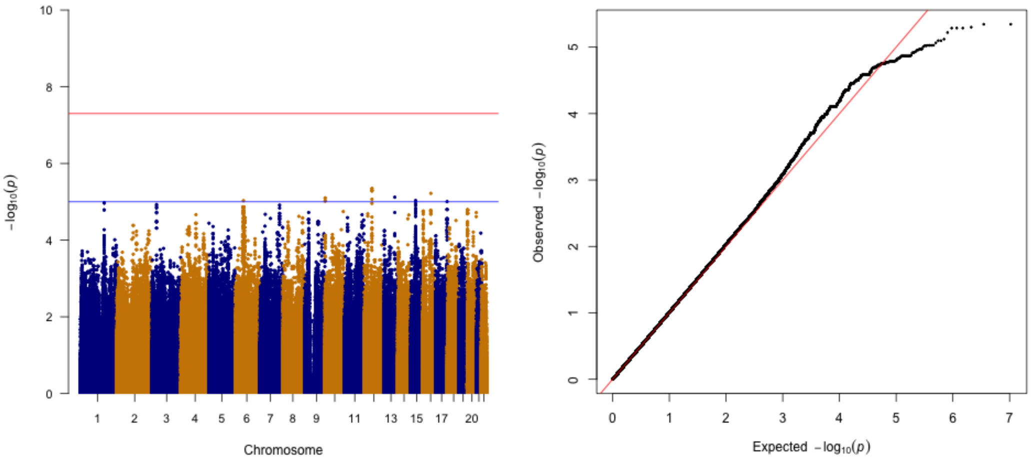

Tired when waking from sleep

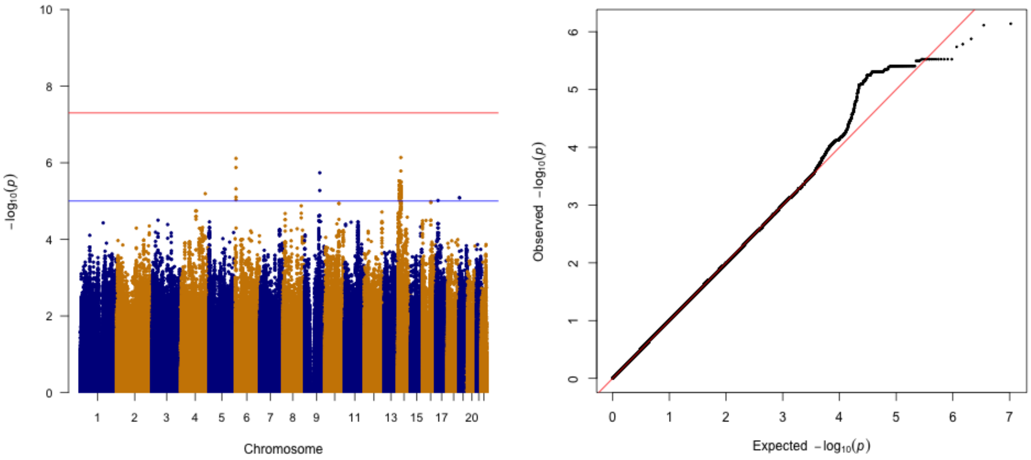

Numbness of limbs when waking from sleep

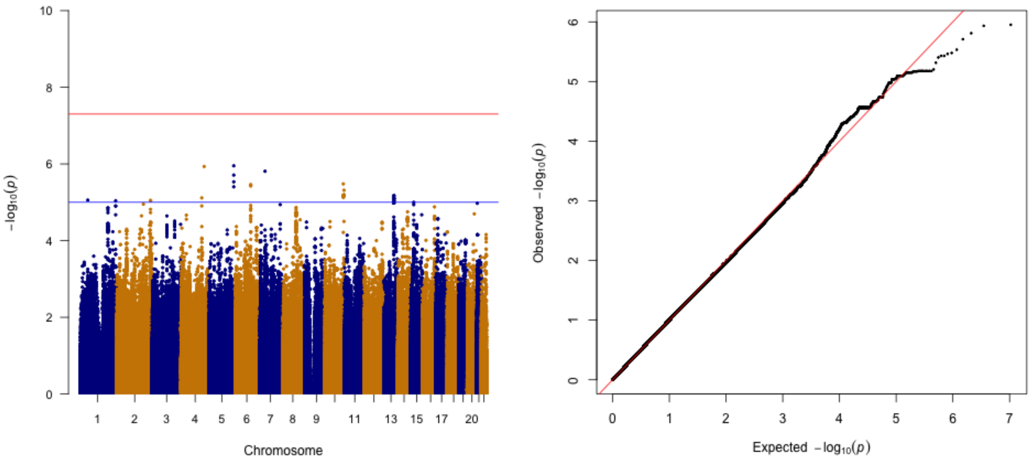

Palpitations and shortness of breath

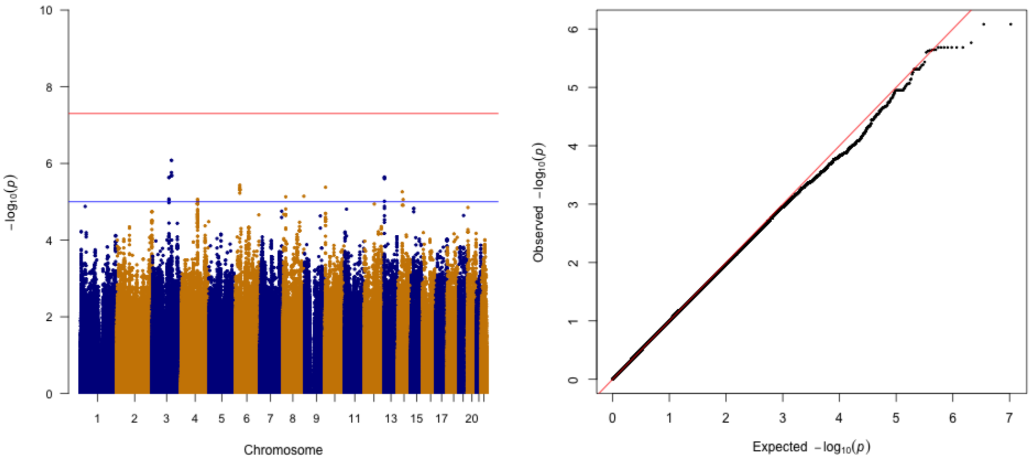

Lower back pain

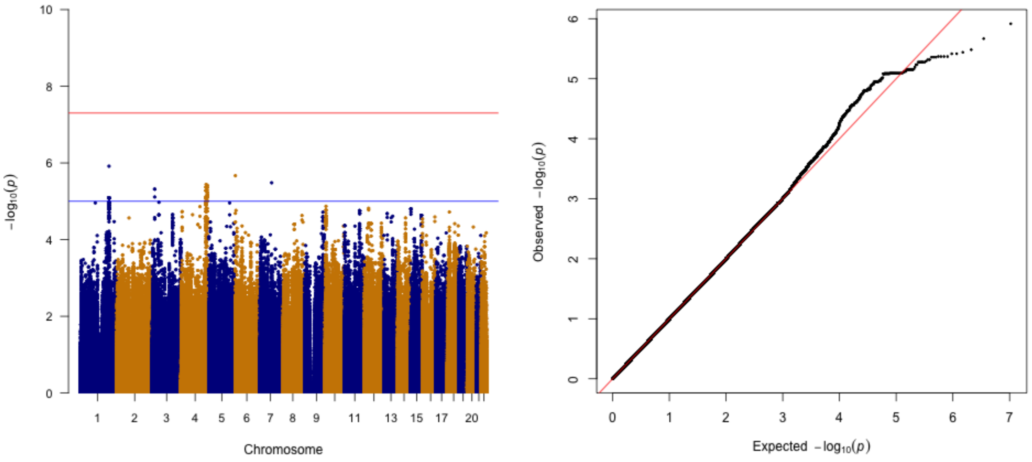

Stiff shoulders

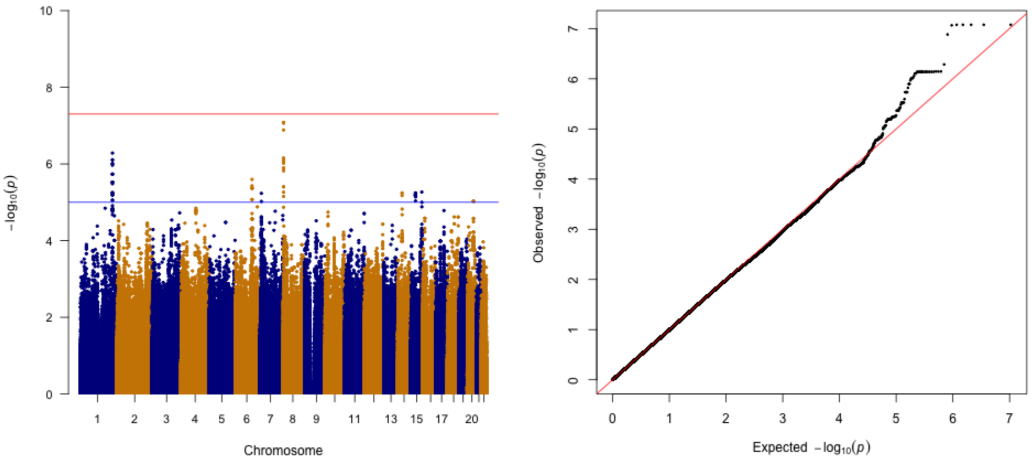

Joint pain

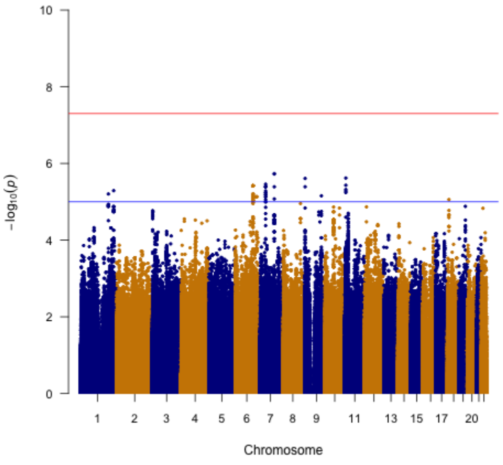

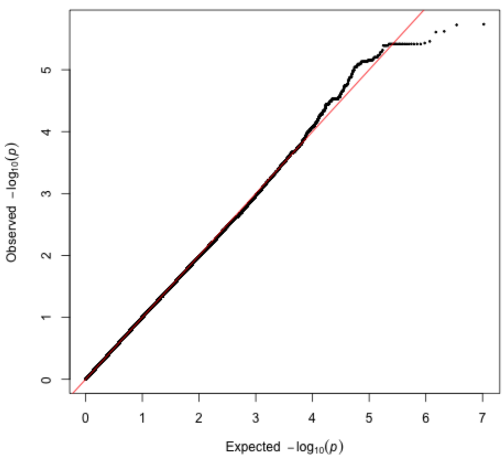

Too lazy to move my body

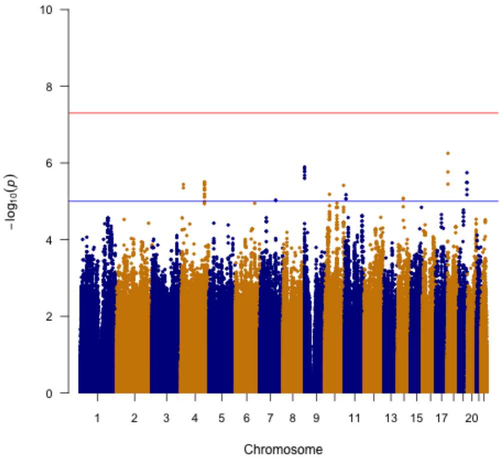

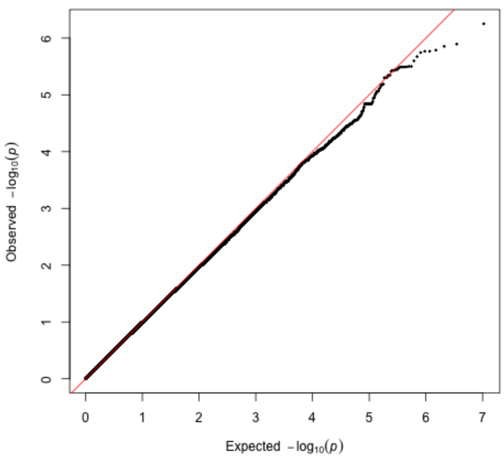

Forgetfulness

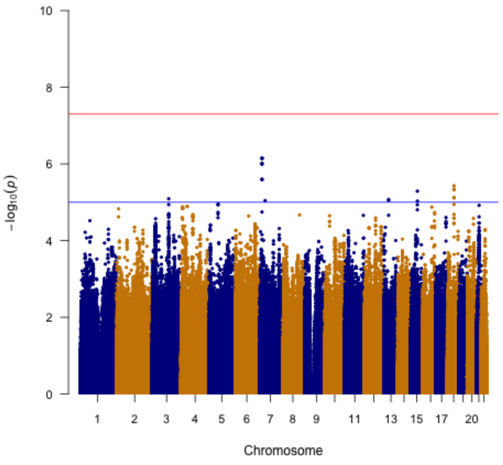

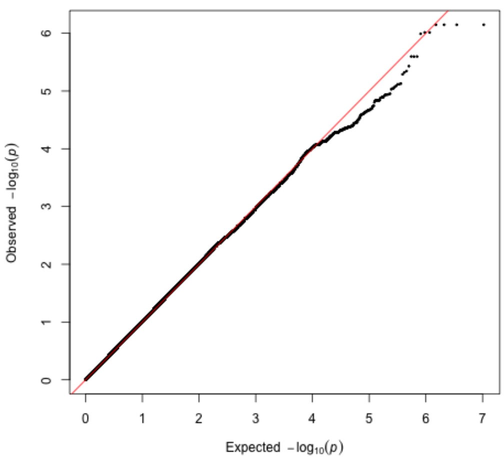

Tendency to gain weight

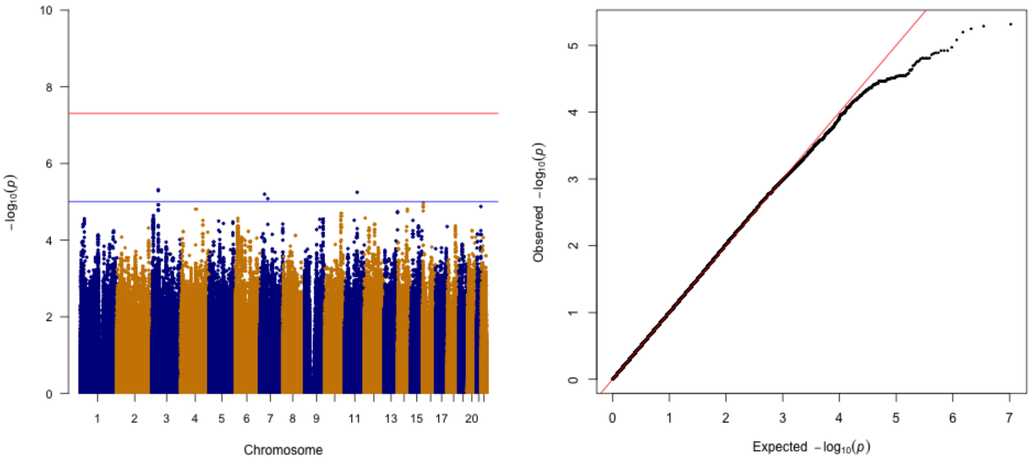

Liven up

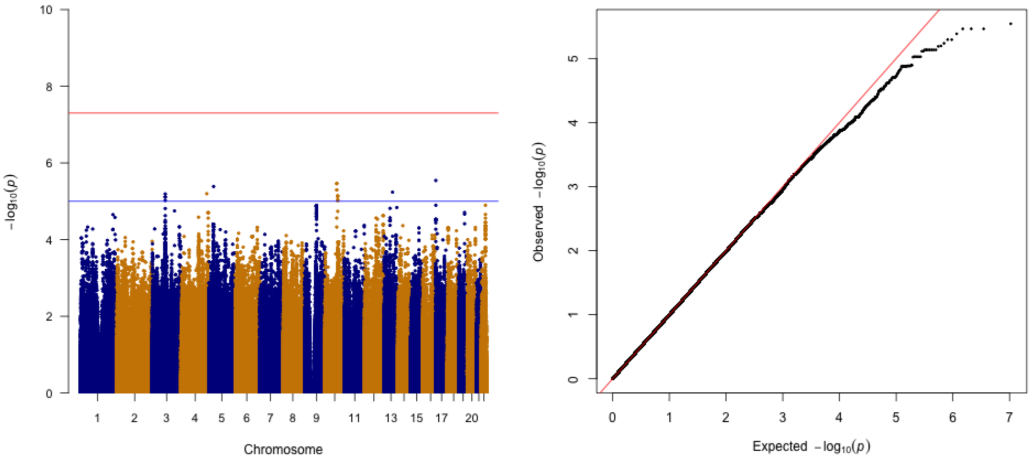

Unwell in the stomach and intestines by stress

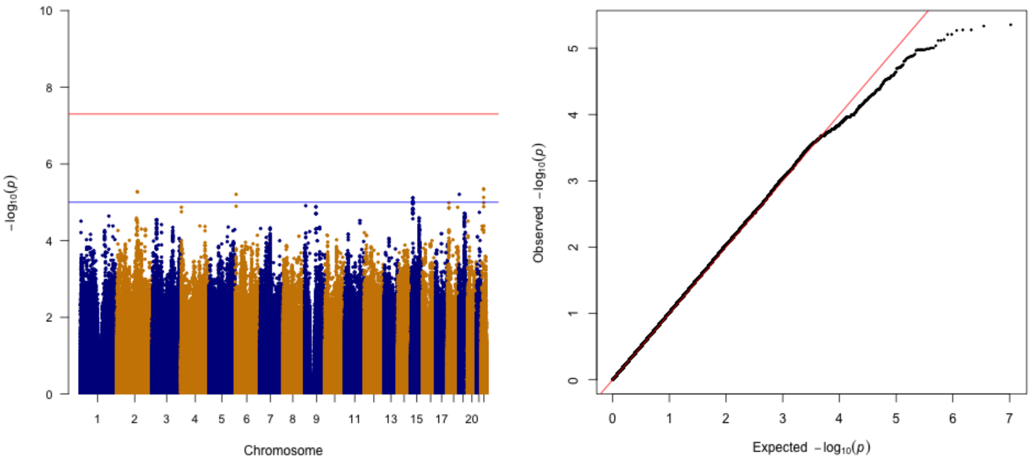

Tendency to think about this and that when asleep

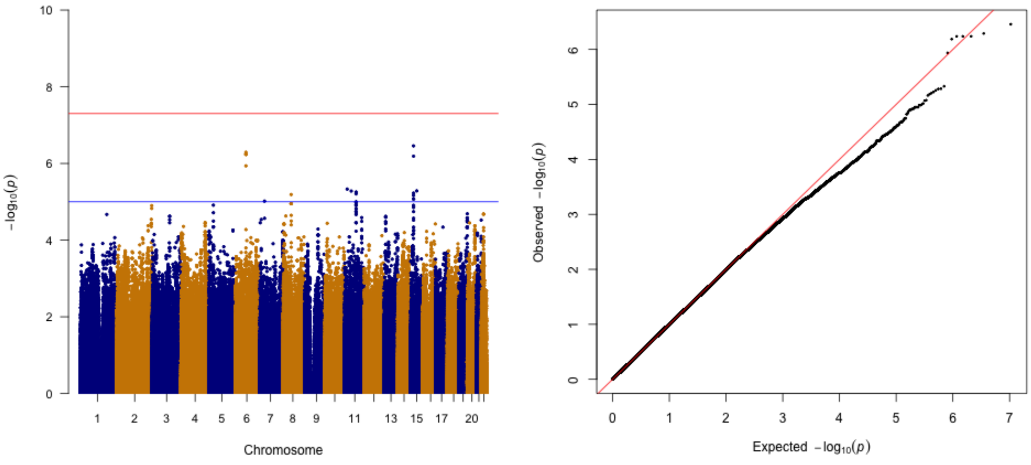

Hard to get thoughts together

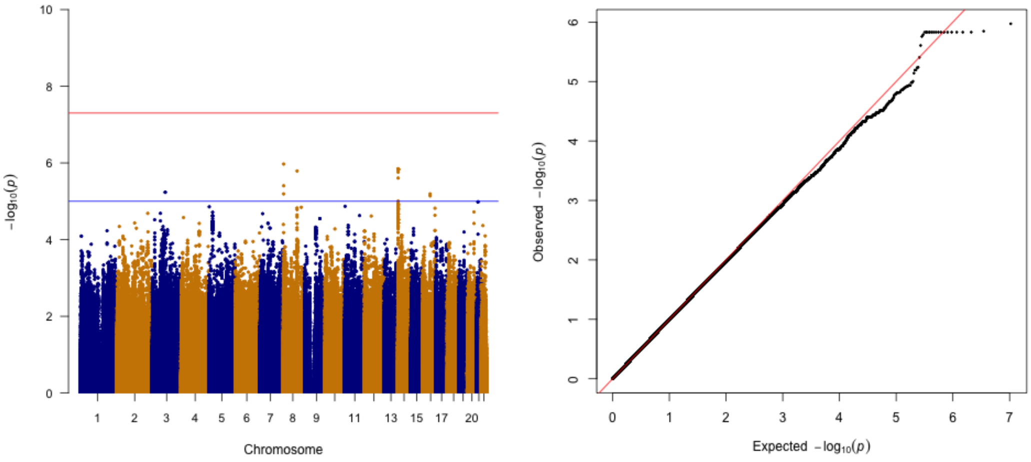

Unable to remember little things

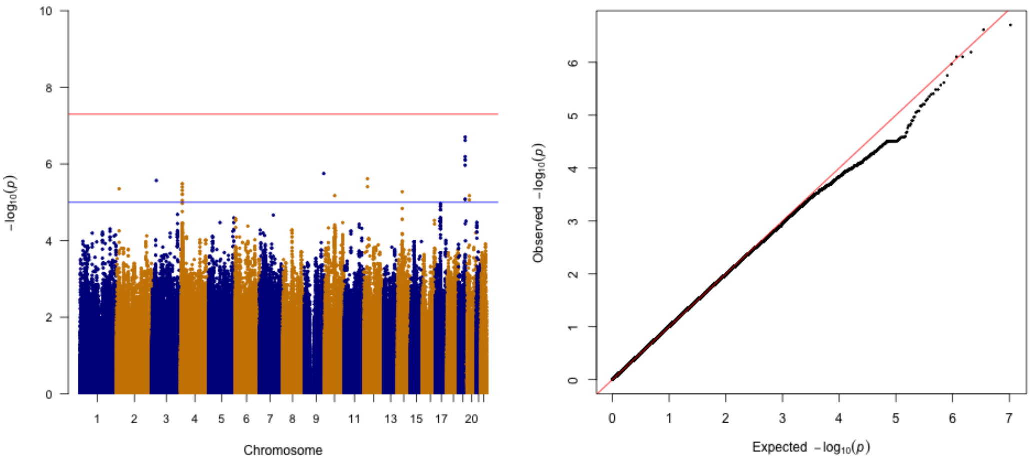

Tendency to make many mistakes

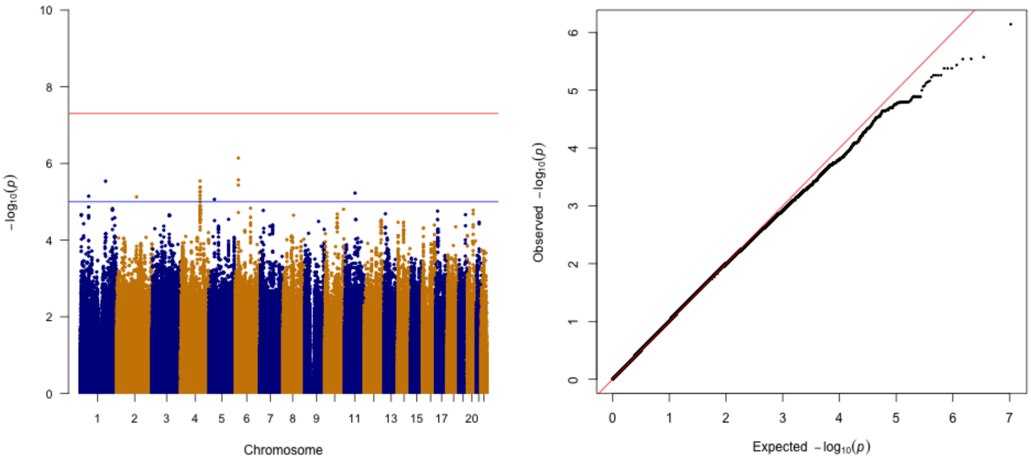

Frequently distracted

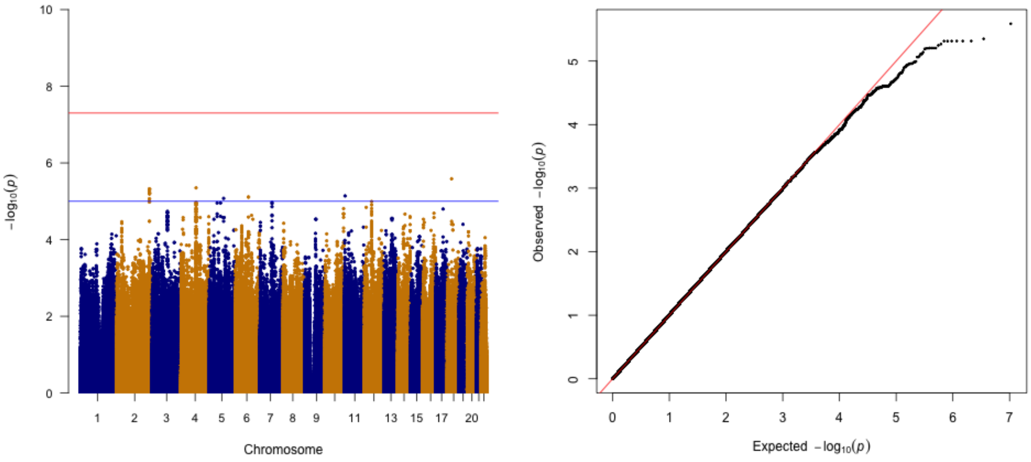

Supplement: S1 Fig — Each trait is indicated in the top left corner of the respective plot. The left plot shows the Manhattan plot, whereas the right plot shows the QQ plot. The red line on the Manhattan plot represents the genome-wide significance level (P < 5E-08), and the blue line represents the genome-wide suggestive significance level (P < 1E-05). (PDF) [file pone.0323778.s005.pdf]
